# Plasma Concentrations of New Biochemical Markers of Atherosclerosis in Patients with Dyslipidemia—A Pilot Study

**DOI:** 10.3390/medicina58060717

**Published:** 2022-05-27

**Authors:** Michał Kosowski, Marcin Basiak, Marcin Hachuła, Bogusław Okopień

**Affiliations:** Department of Internal Medicine and Clinical Pharmacology, Medical University of Silesia, Medyków 18, 40-752 Katowice, Poland; mbasiak@sum.edu.pl (M.B.); marcin.hachula@gmail.com (M.H.); bokopien@sum.edu.pl (B.O.)

**Keywords:** atherosclerosis, dyslipidemia, rupture plaque, osteopontin, osteoprotegerin, metalloproteinase 2, metalloproteinase 9, myeloperoxidase

## Abstract

*Background and Objectives*: The process of atherosclerotic plaque formation and its destabilisation is a process in which many proteins and cytokines are involved. Examples of such proteins are osteopontin (OPN), osteoprotegerin (OPG), metalloproteinases (MMPs) and myeloperoxidase (MPO). The aim of our study is to compare the concentrations of the above-mentioned markers in the plasma of patients with the confirmed presence of rupture plaque in comparison with the plasma of healthy people. *Materials and Methods*: The study included people suffering from dyslipidemia in whom the presence of unstable atherosclerotic plaque was confirmed by ultrasound. The concentrations of OPN, OPG, MPO, metalloproteinase 2 (MMP-2), and metalloproteinase 9 (MMP-9) in the plasma of these people were determined and compared with the concentrations of these proteins in the plasma of healthy people. *Results:* Levels of MMP-2, MMP-9 (*p* < 0.001), OPN, and OPG (*p* < 0.05) were statistically significantly lower in the group of healthy people than in the study group. Differences in MPO concentration were not statistically significant (*p* = 0.073). *Conclusions*: In the plasma of people with confirmed presence of rupture plaque, the concentrations of OPN, OPG, and MMPs are higher compared to the group of healthy people, which may suggest the use of these proteins as novel markers of the presence of unstable atherosclerotic plaque.

## 1. Introduction

One hypothesis is that atherosclerosis is an example of a chronic inflammatory disease. The process of atherosclerotic plaque formation is triggered by an ongoing inflammatory process and involves both endothelial dysfunction and a high level of circulating cholesterol.

It is worth emphasising that not only changes in the concentrations of basic cholesterol fractions allow us to estimate the patients’ cardiovascular (CVD) risk. One such lipoprotein is apolipoprotein B (apoB). A study prepared by Ference et al. indicated that the concentration of apoB correlated more strongly with the risk of atherosclerosis than the concentration of cholesterol [1], which prompted the European Society of Cardiology (ESC) to introduce in the 2019 guidelines a recommendation for determining the concentration of apoB in order to stratify CVD risk [2]. Researchers also observed a strong correlation between the concentration of apolipoprotein (a) (Lp(a)) and CVD risk [3,4], but the lack of standardisation of the methods of its determination resulted in the fact that this is still not used on a large scale.

In the sub-epithelial space of the endothelium damaged by the inflammatory process, foam cells accumulate, thus creating an atherosclerotic plaque, which, as it increases in size, may result in complete occlusion of the artery involved by the atherosclerotic process [5]. Rupture plaque is a separate clinical problem. Such plaque is histopathologically characterised by a large lipid core, low collagen levels, weakened fibrous cap, high macrophage accumulation, and few smooth muscle cells [6]. Due to the high risk of cardiovascular complications, such as strokes and acute coronary syndromes, resulting from the presence of ruptured plaques in the vessels [7,8], it is important to find markers that can assess the risk of such plaques in patients. Such markers include myeloperoxidase, metalloproteinases, osteopontin, and osteoprotegerin.

Myeloperoxidase (MPO) is an enzyme secreted by monocytes found in atherosclerotic plaques and may be involved in the lipid oxidation process [9,10]. Studies using immunohistochemical methods show that the active form of MPO is found only in the endothelium of those blood vessels where the atherosclerotic process takes place [10].

Metalloproteinases (MMPs) are proteins belonging to the endopeptidase family. Their main function in the organism is the damage of the extracellular matrix (ECM), but they are also involved in other processes, such as arterial remodelling [11]. Two of them, metalloproteinase 2 (MMP-2) and metalloproteinase 9 (MMP-9), are also involved in the process of atherosclerotic plaque formation, which is why we can observe higher concentrations in the endothelium of the vessels where the atherosclerotic process takes place [12]. More importantly, some studies show that their increased concentration is correlated with a higher risk of rupture of the atherosclerotic plaque [13].

Due to its multifunctionality, osteopontin (OPN) is a protein found mainly in bone tissue, where it is responsible for the calcification process, but due to its multifunctionality, it can also be found in tissues where the inflammatory process takes place. Because of this, it also plays a role in the process of atherogenesis, especially in the calcification of atherosclerotic plaques [14].

Osteoprotegerin (OPG) is a soluble glycoprotein that has been discovered as an inhibitor of bone resorption, but it is known that it is also active in other tissues, such as smooth muscle and endothelium [15]. Moreover, studies suggest that there is a correlation between the concentration of OPG and the intensity of the inflammatory process within the walls of blood vessels [14].

In our study, we assessed the concentrations of the above-mentioned markers in the serum of patients suffering from dyslipidemia and compared them with the population of healthy people.

## 2. Materials and Methods

The study was carried out on 23 subjects (13 women and 10 men, ranging in age from 28 to 63 years), subdivided into two groups. The study group included 14 patients with the biochemical diagnosis of combined mixed dyslipidemia who did not respond to a 3-month low-fat diet therapy and with confirmed asymptomatic atherosclerosis by the sonographic assessment of common carotid intima-media thickness. Additionally, their anamnesis revealed a familial history of hyperlipidemia among parents or siblings. The control group included nine individuals matched for age and sex with biochemical confirmation of normolipemia. The characteristics of the group can be found in Table 1.

The inclusion criteria for combined mixed dyslipidemia were as follows: total cholesterol (TC) concentration higher than 190 mg/dL; low-density lipoprotein cholesterol (LDL) level higher than 135 mg/dL; and the level of tryglicerides (TG) higher than 150 mg/dL.

The main exclusion criteria in the treated group was the secondary type of lipid disorders (adiposity or overweight regarded as body mass index (BMI) > 27, hypothyreosis, nephrotic syndrome, diabetes mellitus, liver diseases, alcoholism) or other types of dyslipidemia (chylomicronemia or dysbetalipoproteinemia in the forefront). Any other health disorders interfering with serum lipid profile or cytokine serum levels were excluded: any inflammatory diseases, decompensated circulatory failure (NYHA III/IV), unstable angina, myocardial infarction or revascularization intervention within 6 months prior to the beginning of the study; haemodynamically significant cardiac disorders; heart rhythm and conduction system abnormalities; arterial hypertension II° and III°; history of apoplectic stroke episode or transient ischemic attack (TIA), taking drugs interfering with studied substances’ serum levels or affecting lipid metabolism (e.g., hypolipidemic drugs, niacin, non-selective β-blockers) within 3 months before starting the study. Patients treated with anticoagulants were also excluded.

Apart from the patient’s history and physical examination, the following tests were performed: total blood count, red blood cell count, leucocyte differential count, erytrocyte sedimentation rate, glycated hemoglobin, liver enzyme test, plasma protein and lipoprotein electrophoresis, urine analysis, abdomen ultrasonography, electrocardiography, fasting serum level, and specialist consultations if needed. The additional tests were performed if required: creatinine, aminotransferase, bilirubin, platelet count, hematocrit, white blood cell count, or pregnancy test. The abovementioned procedures were conducted to accurately enroll patients in the study.

Venous blood samples from both groups were collected after an overnight 12 h fasting at 8 a.m. All the tests were carried out by a person blinded to the subject’s identity and all clinical details. Plasma lipids were assayed by routine laboratory techniques. LDL levels were measured directly. The plasma levels of MMP-2, MMP-9, MPO, OPN and OPG were assessed by commercially available enzyme immunoassay methods using Cloud-Clone Corp. USA; Diaclone, France; Immundiagnostik AG, Germany and Bio-Vendor, Czech Republic kits as described by the manufacturer. All the laboratory tests were also performed in the control group. To avoid the freeze-thawing effect, each assay was performed on a single sample aliquot.

The study was approved by the Ethical Committee of the Medical University of Silesia. All the study participants signed an informed consent form.

### 2.1. Specific Inclusion Criteria for Atherosclerotic Plaque at High Risk

Ultrasonography is a method for evaluating atherosclerotic carotid disease; it is used clinically to evaluate the presence of plaque, the degree of carotid stenosis using blood-flow velocity profiles, and the carotid intima-media thickness. Examining the carotid arteries and determining the complex intima media thickness (C-IMT) in the extracranial region using B-mode ultrasound with a linear probe at 7.5–10 MHz frequency. The C-IMT was assessed three times, and the average result was considered. The measurement was made in the distal common carotid artery (1 cm proximal to the carotid bulb). As previously described [16], unstable plaques were associated with fibrofatty and hemorrhagic content and an echolucent appearance. Moreover, high-risk plaques may have an irregular surface or ulcerations, which were discovered using the Color–Doppler technique [17].

### 2.2. Statistical Analysis

The data were processed using Statistica TIBCO Software Inc., Palo Alto, CA, USA, (2017) version 13.3 software, which was licensed by the Medical University of Silesia in Katowice. To assess the normality of distributions, we used the Shapiro-Wilk test. To compare quantitative variables, the t-test for independent means was used. Additionally, we used the U. Mann-Whitney test in the case of non-compliance with the condition of t-test. We also use Spearman’s rank correlation to assess the relationship between variables. We assumed that a *p*-value of less than 0.05 was statistically significant.

## 3. Results

In the study group, no significant differences in demographic data (age, gender, smoking, and weight) were observed.

In the control group, we observed statistically significantly lower concentrations of TC, LDL, non high-density lipoprotein cholesterol (non-HDL) and TG (*p* < 0.001) than in the study group. High-density lipoprotein cholesterol (HDL) levels, on the other hand, showed no statistically significant differences (*p* = 0.47).

In our control group, the levels of OPN (*p* < 0.05) and OPG (*p* < 0.05) were also statistically significantly lower than in the study group. We observed the same correlation in the case of MMP-2 and MMP-9 concentrations (*p* < 0.001). Opposite observations were made in the case of MPO concentration, which was higher in the control group compared to the study group, but this correlation was not statistically significant (*p* = 0.073).

Detailed results are presented in Table 2 and Figure 1, Figure 2, Figure 3, Figure 4, Figure 5, Figure 6, Figure 7 and Figure 8.

We have also verified the relationship between the concentrations of examined markers and individual cholesterol fractions, proving the presence of a negative correlation between the concentration of OPN and TC, LDL, TG, and non-HDL and a positive correlation between the concentration of OPG and TC, LDL, and non-HDL. Such a correlation was not observed between interleukin concentrations and HDL levels. Detailed results are presented in Table 3.

## 4. Discussion

Year by year, atherosclerosis and its complications in the form of cardiovascular events are an increasingly frequent cause of death [18]. It is for this reason that research into such biochemical parameters has begun, which will allow for even better estimation of the risk of a cardiovascular event in patients than the lipid profile determinations used thus far. The researchers’ attention is particularly focused on proteins that may be responsible for the stabilisation of atherosclerotic plaque, and thus influence this risk.

In our study, we proved that in the group of people not suffering from lipid disorders, in whom the presence of plaque rupture was confirmed by ultrasound methods, we could observe different concentrations of proteins such as OPG, OPN, MMP-2, MMP-9, and MPO compared to the group of healthy people, in which proteins are involved in the formation and stabilisation of atherosclerotic plaque.

The first reports, carried out in animal models, indicating the important role of OPG in the process of atherosclerotic plaque formation and their destabilization, appeared at the beginning of the 21st century [19]. These assumptions were confirmed in numerous studies involving patients with diagnosed symptomatic atherosclerosis [20,21,22]. The largest of them was carried out on a group of almost 6,000 patients by Mogelvang R. et al. and confirmed the relationship between the concentration of OPG, the risk factors of atherosclerosis and clinical atherosclerosis described so far in the literature [23]. In recent years, there have also been reports clearly confirming the relationship between the concentration of OPG and the appearance of unstable atherosclerotic plaques in arteries [24]. However, they were carried out on a population of patients with a significant risk factor for cardiovascular diseases, which was type 2 diabetes.

Similar observations regarding the influence of atherosclerotic plaques on the stabilisation process of atherosclerotic plaques were made by the researchers in relation to OPN. In 1994, Shanahan C.M et al. noticed the high expression of OPN within atherosclerotic plaques, postulating its influence on the process of plaque calcification [25]. Conducted much later, in 2018, two studies comparing the concentration of OPN within atherosclerotic plaques in people with symptomatic and asymptomatic atherosclerosis gave different results. One of them by Guo Z.Y. et al. indicates that within unstable atherosclerotic plaques, the concentration of OPN is significantly lower compared to its concentration in stable plaques [26]. The second, conducted by Kyriakidis K. et al. proves that the concentration of OPN within atherosclerotic plaques in people diagnosed with symptomatic atherosclerosis is higher than in people with asymptomatic atherosclerosis [27]. At the moment, however, there are no reports assessing the plasma concentration of OPN in patients with the confirmed presence of unstable atherosclerotic plaque.

An interesting topic is still the correlation between the concentration of cholesterol fractions and OPN and OPG, which was proven in our study. The same correlation between the concentration of OPG and LDL and TC was observed in 2005 by Eun et al. hypothesising that it may be related to the protective role of OPG in preventing the formation of atherosclerotic plaques [28]. As for OPN, Luomala et al. observed a negative correlation between the concentration of OPN and the lathosterol produced within the muscles [29]. Although the mechanism of this phenomenon is not fully understood, it is consistent with in vitro studies in which inhibition of 3-Hydroxy-3-methylglutaryl-coenzyme A reductase with simvastatin, and thus a decrease in cholesterol production within cells, resulted in an enhancement in OPN levels [30].

A topic that has been much better studied by scientists is the relationship between the plasma concentration of MMPs and the presence of rupture plaques. Due to their mechanism of action consisting of remodelling and degradation of the ECM [31], they were among the first to be postulated as important in the process of atherosclerotic plaque formation. Many studies clearly confirm that high concentrations of MMP-2 and MMP-9 in the plasma correlate with the presence of unstable atherosclerotic plaques within the blood vessels, and thus with a higher risk of a cardiovascular event in patients [32,33,34,35]. For this reason, investigators postulate their use as novel, independent markers indicating the presence of unstable atherosclerotic plaque [36]. These results are in line with the fact that we found statistically significantly lower levels of MMP-2 and MMP-9 in this group of people in our study who did not have unstable atherosclerotic plaque.

Another protein we study, MPO, is very strongly related to MMPs and their influence on atherosclerotic plaque. Taking into account its mechanism of action, which is the activation of MMPs and the inactivation of inhibitors of these proteins, it would seem logical that this protein, like MMPs, is associated with the process of destabilisation of atherosclerotic plaques. Moreover, MPO-generated oxidants are able to deactivate the metaloproteinase-1 inhibitor, which leads to an increase in the concentration of this protein and an even more intense degradation of the ECM [37]. That is why the information unexpected by scientists turned out to be the results of the research presented by Brennan M.L. et al., who observed that in MPO-deficient mice, the severity of atherosclerotic lesions was much greater than in the control group, whose MPO concentration was normal [38]. This observation seems to be consistent with the higher MPO concentration observed in our group of healthy people compared to the group of people suffering from atherosclerosis. However, it should be remembered that in our study, the differences between the two groups were not statistically significant. On the other hand, many studies show that in patients diagnosed with unstable coronary artery disease, plasma MPO concentrations are much higher than in control groups, and this would suggest an inverse relationship between MPO concentration and the presence of rupture plaques in arteries [39,40,41].

To the best of our knowledge, there are still no studies that would compare the concentrations of the above-mentioned proteins in the group of patients with subclinical or asymptomatic atherosclerosis and dyslipidemia with their concentrations in the group of healthy people. Markers presented in our study should therefore be considered as modern markers of the presence of unstable atherosclerotic plaques in the arteries. However, it should be taken into account that when searching for any single biomarker, a certain percentage of people with abnormal levels will not have the disease (false positive results), while those who are ill may have normal levels (false positive results). Therefore, in the future, biomarker panels and index scores will be useful and could be used in medicine to improve diagnosis and aid in prognosis. Additional studies on larger groups of patients are needed to unambiguously assess this relationship.

Our investigation has several limitations. First, the duration of the trial was limited, and clinical outcomes, such as the occurrence of cardiovascular events over extended follow-up, were not evaluated. Secondly, one of the limitations of our study is the lack of an additional control group of patients with stable atherosclerotic plaque. The presence of such group would allow more precise assessment of the relationship between the markers studied by us and the unstability of the atherosclerotic plaque. In addition, although the study population exceeded the minimum sample size, it was still quite small. However, this is because the number of patients treated with proprotein convertase subtilisin/kexin 9 (PCSK9) inhibitors in our nation is very low, and we have chosen very specific and rigorous criteria to meet. Lastly, our study did not include type 2 diabetic subjects; therefore, more research is required to determine whether a comparable favourable effect on monocyte secretory function and systemic inflammation is also found in diabetic patients.

## 5. Conclusions

In the group of patients with dyslipidemia and the confirmed presence of unstable atherosclerotic plaque, plasma levels of OPG, OPN, MMP-2, and MMP-9 are significantly higher compared to the group of healthy people. These proteins should therefore be considered as modern markers of the presence of unstable atherosclerotic plaques in the arteries. However, additional studies on larger groups of patients are needed to unambiguously assess this relationship.

## Figures and Tables

**Figure 1:**
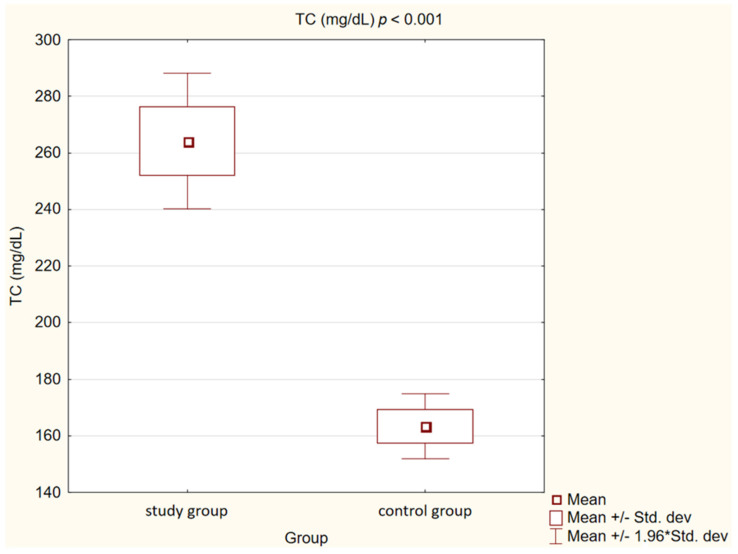
Concentration of total cholesterol (TC) in study and control group.

**Figure 2:**
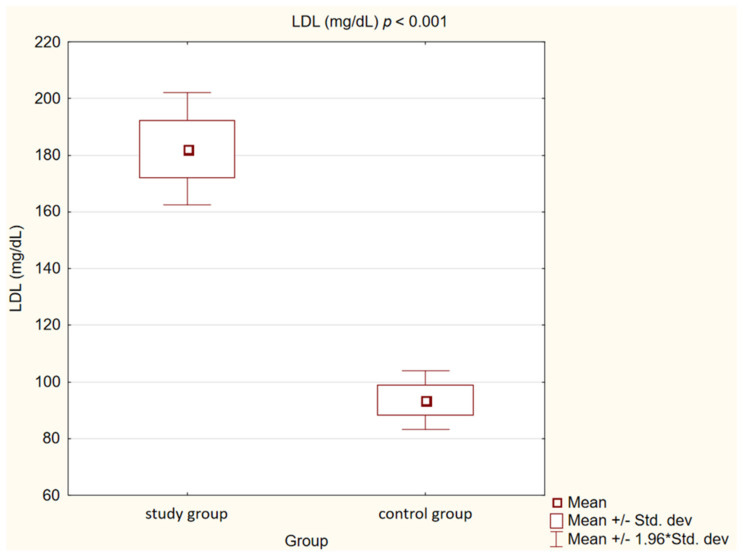
Concentration of low-density lipoprotein cholesterol (LDL) in study and control group.

**Figure 3:**
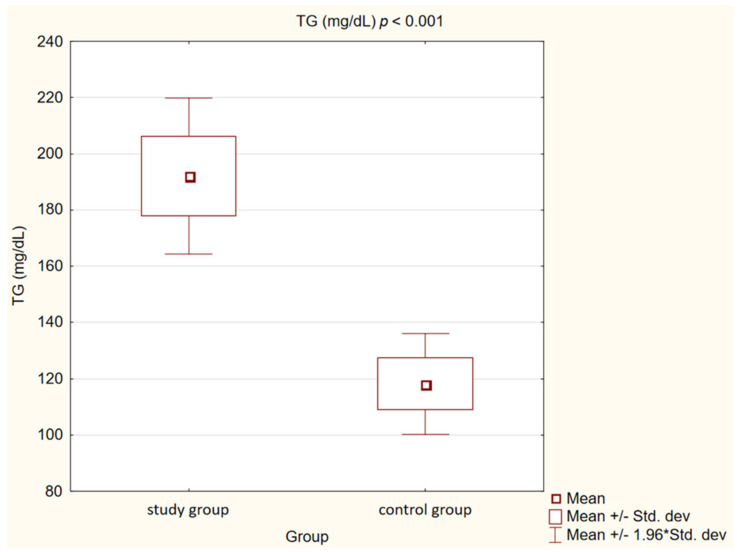
Concentration of triglycerides (TG) in study and control group.

**Figure 4:**
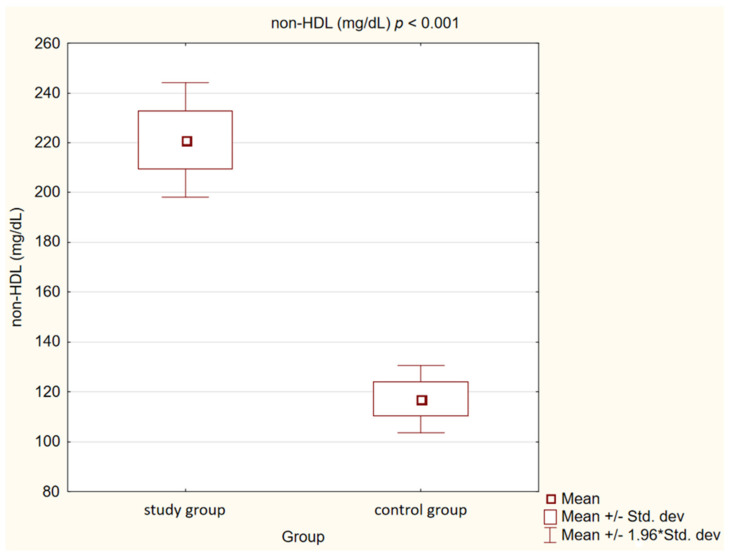
Concentration of non high-density lipoprotein cholesterol (non-HDL) in study and control group.

**Figure 5:**
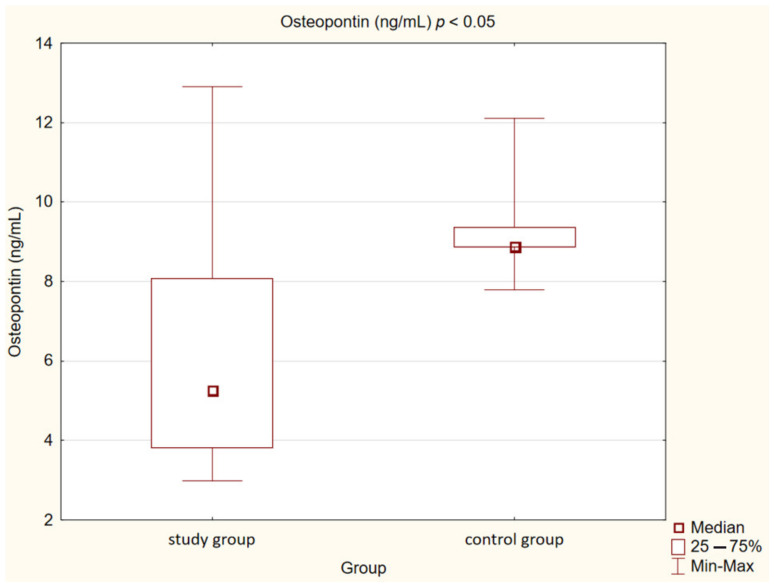
Concentration of osteopontin (OPN) in study and control group.

**Figure 6:**
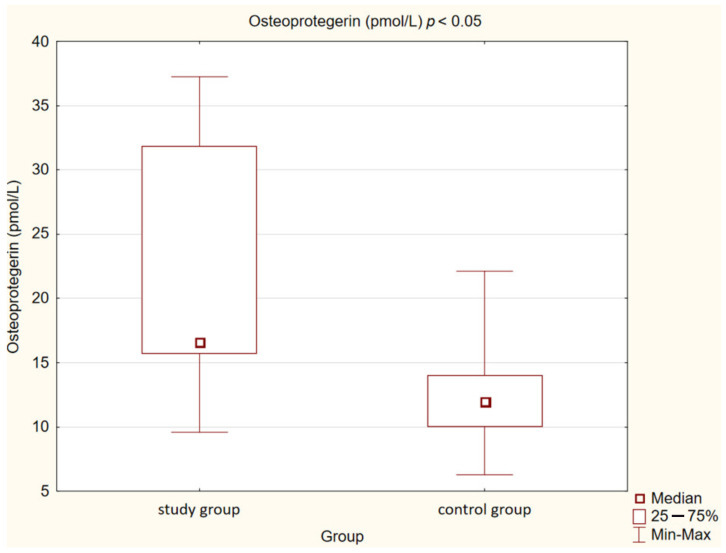
Concentration of osteoprotegerin (OPG) in study and control group.

**Figure 7:**
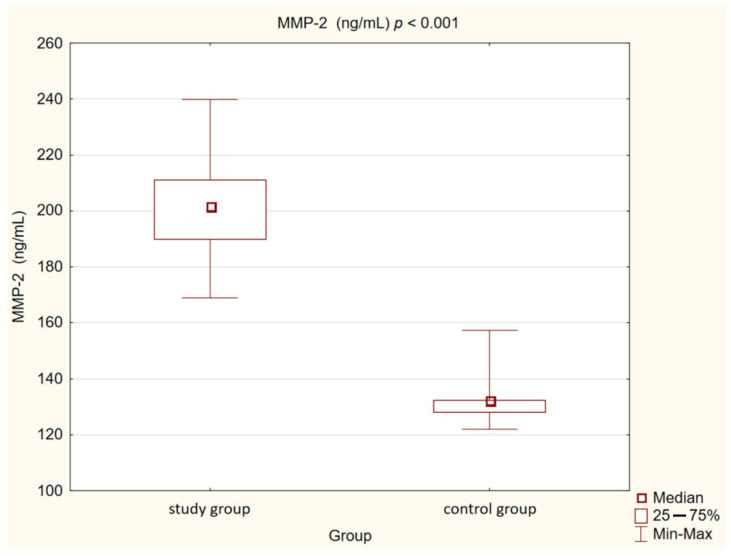
Concentration of metalloproteinase 2 (MMP-2) in study and control group.

**Figure 8:**
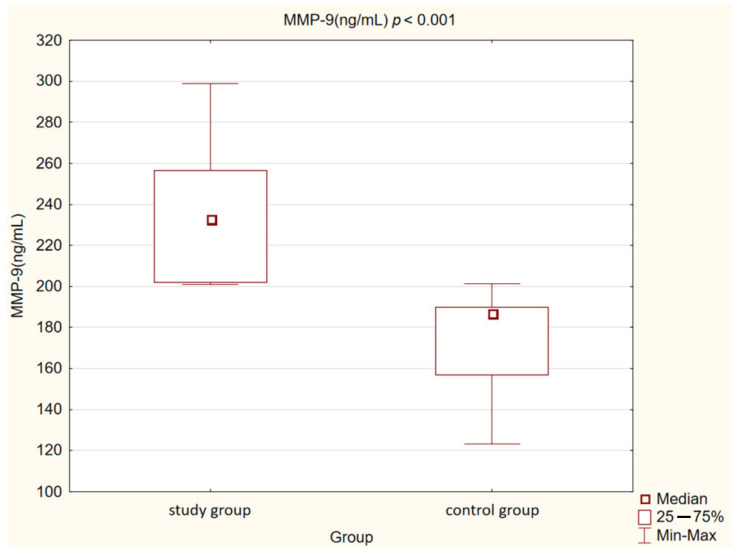
Concentration of metalloproteinase 9 (MMP-9) in study and control group.

**Table 1:** Baseline characteristics of patients (values are mean ± SD).

	Control Group	Study Group
Number of patients	9	14
Age, years	43 ± 5	45 ± 6
BMI	27.8 ± 2.4	27.4 ± 2.3
Smokers, %	33.3	35.7
Systolic blood pressure, mmHg	131 ± 7	132 ± 6
Diastolic blood pressure, mmHg	81 ± 3	82 ± 4
Fasting glucose, mg/dL	91 ± 4	92 ± 5

**Table 2:** Cholesterol fraction and cytokine levels in the study and control group. Q1 is the first quartile; Q3 is the third quartile.

	Study Group			Control Group			*p* Value
Total cholesterol (mg/dL)	264.24 ± 38.55			163.44 ± 17.65			<0.001
Low-density lipoprotein cholesterol (mg/dL)	182.22 ± 32.02			93.51 ± 15.87			<0.001
High-density lipoprotein cholesterol (mg/dL)	43.09 ± 12.26			46.31 ± 5.09			0.47
Non-high-density lipoprotein cholesterol (mg/dL)	221.15 ± 37.07			117.13 ± 20.62			<0.001
Triglycerides (mg/dL)	192.01 ± 44.78			118.18 ± 27.5			<0.001
Myeloperoxidase (ng/mL)	353.19 ± 175.92			470.52 ± 74.6			0.073
	Median	Q1	Q3	Median	Q1	Q3	
Osteopontin (ng/mL)	5.27	3.81	8.08	8.9	8.88	9.36	<0.05
Osteoprotegerin (pmol/mL)	16.62	15.74	31.86	11.99	10.02	14	<0.05
Metalloproteinase 2 (ng/mL)	201.64	189.9	211.1	132.2	128.1	132.2	<0.001
Metalloproteinase 9 (ng/mL)	232.88	201.9	256.5	187.1	156.8	189.9	<0.001

**Table 3:** Correlation between cholesterol fraction levels and levels of osteopontin (OPN) and osteosteoprotegerin (OPG).

	Osteopontin Concentration	Osteoprotegerin Concentration
Total cholesterol level	R = −0.63, *p* < 0.01	R = 0.52, *p* < 0.05
Low-density lipoprotein cholesterol level	R = −0.62, *p* < 0.01	R = 0.56, *p* < 0.05
High-density lipoprotein cholesterol level	R = 0.1, *p* > 0.05	R = −0.02, *p* > 0.05
Non-high-density lipoprotein cholesterol level	R = −0.59, *p* < 0.01	R = 0.52, *p* < 0.05
Triglycerides level	R = −0.45, *p* < 0.05	R = 0.38, *p* > 0.05

## Data Availability

The data that support the findings of this study are available from one of the authors (mbasiak@sum.edu.pl) on reasonable request.
